# Clinical Application of the New Prostate Imaging for Recurrence Reporting (PI-RR) Score Proposed to Evaluate the Local Recurrence of Prostate Cancer after Radical Prostatectomy

**DOI:** 10.3390/cancers14194725

**Published:** 2022-09-28

**Authors:** Federica Ciccarese, Beniamino Corcioni, Lorenzo Bianchi, Antonio De Cinque, Alexandro Paccapelo, Giovanni Luca Galletta, Riccardo Schiavina, Eugenio Brunocilla, Rita Golfieri, Caterina Gaudiano

**Affiliations:** 1 Department of Radiology, IRCSS Azienda Ospedaliero Universitaria di Bologna, 40138 Bologna, Italy; 2 Division of Urology, Department of Urology, IRCCS University Hospital of Bologna, 40138 Bologna, Italy

**Keywords:** multiparametric magnetic resonance imaging, prostate cancer recurrence, radical prostatectomy

## Abstract

**Simple Summary:**

The aim of the new Prostate Imaging for Recurrence Reporting (PI-RR) is a standardization in reporting to assess the likelihood of relapse after radical prostatectomy. Our study documented an excellent inter-observer agreement in recurrence reporting when using the PI-RR score, demonstrating a wide reproducibility, thus supporting the wide use of the PI-RR score in the clinical practice. The diagnostic accuracy was 68.4%, with the detection rate influenced by the PSA values. Overall, the PI-RR score globally showed a higher detection rate than PET/CT scans for local recurrence.

**Abstract:**

Background: We investigated the diagnostic accuracy of the new Prostate Imaging for Recurrence Reporting (PI-RR) score and its inter-observer variability. Secondly, we compared the detection rate of PI-RR and PET and analyzed the correlation between Prostate Specific Antigen (PSA) levels and the PI-RR score. Methods: We included in the analysis 134 patients submitted to multiparametric magnetic resonance imaging for suspected local recurrence. The images were independently reviewed by two radiologists, assigning a value from 1 to 5 to the PI-RR score. Inter-observer agreement and diagnostic accuracy of the PI-RR score (compared to histopathological data, available for 19 patients) were calculated. The detection rate was compared to those of choline PET/CT (46 patients) and PSMA PET/CT (22 patients). The distribution of the PSA values in relation to the PI-RR scores was also analyzed. Results: The accuracy of the PI-RR score was 68.4%. The reporting agreement was excellent (K = 0.884, *p* < 0.001). The PI-RR showed a higher detection rate than choline PET/CT (69.6% versus 19.6%) and PSMA PET-CT (59.1% versus 22.7%). The analysis of the PSA distribution documented an increase in the PI-RR score as the PSA value increased. Conclusion: The excellent reproducibility of the PI-RR score supports its wide use in the clinical practice to standardize recurrence reporting. The detection rate of PI-RR was superior to that of PET, but was linked to the PSA level.

## 1. Introduction

Radical prostatectomy (RP) is a common treatment choice in patients with localized prostate cancer suitable for surgery [1,2]. However, 27–53% of patients who undergo primary intended curative therapy develop a biochemical recurrence (BCR), depending on their preoperative risk and stage of cancer [3]. A confirmed rising level of prostate-specific antigen (PSA) is considered a sign of disease recurrence; there is no specific cutoff to define BCR, but PSA is expected to be undetectable within 2 months after a successful RP [1]. As a PSA level ≥ 0.4 ng/mL is predictive of metastatic disease, this value is considered a threshold to report the outcome of RP [4].

The PSA level alone does not differentiate a local relapse from a metastatic disease; however, the pattern of its rise has been included into clinical nomograms to predict whether recurrence is more likely to be local or systemic, in the attempt to optimize management and salvage treatment [5]. Of the imaging modalities, multiparametric magnetic resonance imaging (mpMRI) is reported to be superior to choline positron emission tomography/computed tomography (PET/CT) in the detection of local recurrence [6]. Recently, a great interest has been developing in prostate-specific membrane antigen (PSMA) PET-CT, but its usefulness has not been completely defined, especially in patients with a low PSA level (<0.5) [7].

With the aim of standardizing image acquisition and reporting of mpMRI, a panel of experts from the European Society of Urogenital Radiology and the European Society of Urologic Imaging and individual members of the Prostate Imaging-Reporting and Data System (PI-RADS) Steering Committee proposed a Prostate Imaging for Recurrence Reporting (PI-RR) score to assess the likelihood of relapse [8]. A recent paper by Pecoraro et al. demonstrated an excellent inter-observer agreement in recurrence reporting (interclass correlation coefficient of 0.87), with a high diagnostic accuracy, ranging from 75 to 85% (depending on the reader) that could allow a wide use of the PI-RR score in the clinical practice. However, the authors claimed that several clinical issues must still be considered: first, the results were obtained from a single-center study based on a small sample size, requiring additional validation in further studies to be considered definite; secondly, the roles of MRI and PET/CT need to be compared [9].

Taking all this into account, we evaluated the diagnostic accuracy of the proposed PI-RR score (compared to histopathological data) and the inter-observer variability in recurrence reporting, in order to compare our results to those previously reported. Secondly, we evaluated the detection rate of mpMRI and PET-CT (choline and PSMA) for local recurrence and the correlation between the PSA level and the PI-RR score.

## 2. Materials and Methods

### 2.1. Study Population

This study was a retrospective and single-center study, approved by our local institution review board and conducted in accordance with the Declaration of Helsinki.

We included 151 patients with clinical suspicion of local recurrence after RP who were submitted to mpMRI at our Institute from March 2010 to March 2022. 

The exclusion criteria were: (1) mpMRI protocol not completely adhering to the suggested imaging protocol described in PIRADS version 2.1 [10]; (2) evidence of systemic recurrence or local recurrence outside the prostatic bed (pelvic nodes) on any imaging modality.

Overall, 3 patients were excluded because dynamic-contrast enhancement (DCE) imaging was not performed, 7 patients were excluded for extra-pelvic recurrence, 7 patients were excluded for pelvic nodal recurrence.

A total of 134 patients were considered for the final analysis.

For each patient, we collected the following clinical data:

- PSA level (available in 109 patients)
- Results of PET-CT (46 patients were submitted to choline PET-CT, and 22 to Ga-PSMA PET-CT within one month from mpMRI) that were dichotomized as positive or negative depending on the clinical report
- Results of transrectal ultrasonography (TRUS)-guided biopsy within 3 months from mpMRI (19 patients)
- Histopathological data of the primary tumor were collected, when available (39 patients).

### 2.2. mpMRI Protocol Study

The mpMRI examinations were performed using a 1.5 T whole-body scanner (Signa HDxt; GE Healthcare, Milwaukee, WI, USA) and a standard 8-channel pelvic phased-array surface coil combined with a disposable endorectal coil.

The protocol study included:

- Morphological study: Fast Relaxation Fast Spin Echo T2-weighted (T2w) sequences in the sagittal, axial and coronal planes, covering the prostate lodge.
- Diffusion-weighted imaging (DWI): a single-shot echo-planar sequence with a high b-value (2000 s/mm^2^) and another single-shot echo-planar sequence with two different b-values (50 and 1000 s/mm^2^), this latter for the calculation of the apparent diffusion coefficient (ADC) map.
- DCE acquisition: three-dimensional (3D) T1-weighted Time-of-Flight Spoiled Gradient-Recalled sequence on the axial plane during the intravenous injection of a gadolinium-based contrast agent at a flow rate of 3 mL/sec followed by 15 mL of saline solution. The 3D data sets were acquired with a 10 s temporal resolution; the acquisitions before the contrast agent administration were analyzed to detect foci of hemorrhage.

Both the DWI and the DCE images were obtained on axial plane, with the same parameters as the T2w axial sequence, in order to obtain a match; moreover, the DWI and DCE images were processed on an independent workstation with dedicated software (Functool, 4.5.5, GE Healthcare, Milwaukee, WI, USA).

### 2.3. Image Analysis

All the mpMRI images were independently reviewed by two radiologists with, respectively, 10 years (B.C.) and 5 years (F.C.) of experience in mpMRI; the readers were not blinded to the clinical and pathological data (including PSA level and primary tumor location).

**Figure 1:**
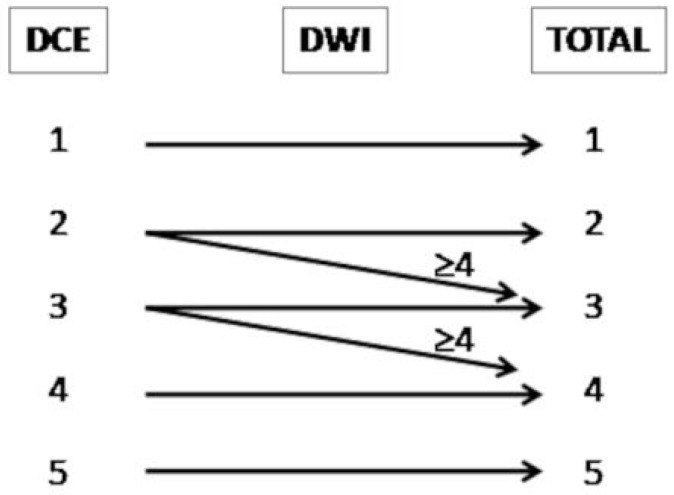
PI-RR score assessment.

A score from 1 to 5 was assigned to all mpMRI scans according to the PI-RR proposal [5]. The score assessment is summarized in Table 1 and Figure 1.

- Scores of 1 and 2 were assigned to lesions with a very low and low likelihood of recurrence
- Score 3 was assigned if the presence of recurrence was uncertain
- Scores 4 and 5 were assigned when the likelihood of recurrence was high and very high

### 2.4. Statistical Analysis

Data are presented as means ± standard deviations, ranges, and frequencies. The diagnostic performance of the PI-RR score was evaluated with sensitivity, specificity, positive predictive value (PPV), negative predictive value (NPV) and accuracy (correctly classified cases) compared to biopsy; mpMRI was considered positive when the PI-RR score was ≥3, as previously reported [9]. Non-parametric correlations were evaluated with Spearman’s Rho (ρ). Mann–Whitney U and Fisher’s Exact tests were used when appropriate. Receiver-operating characteristics (ROC) curves were plotted. The area under the ROC curve (AUC) was computed together with the 95%CI and the asymptotic test for null hypothesis: true area = 0.5. The best cutoff for PSA was calculated using the maximization of the Youden’s Index. Cohen kappa was used to evaluate the inter-reader agreement. All tests were 2-tailed, and a *p* value of <0.05 was considered statistically significant. All statistical analyses were carried out using IBM SPSS 25.0 (SPSS Inc., Armonk, NY, USA).

## 3. Results

### 3.1. Diagnostic Accuracy of the PI-RR Score and Inter-Observer Agreement

The mean age of the patients was of 69.2 years (±6.6). Overall, 19 patients underwent a TRUS-guided biopsy of the vesico-urethral anastomosis; 13 biopsies were positive for local recurrence, and 6 were negative. The PI-RR score distribution in relation to the biopsy results is shown in Table 2.

Compared to biopsy, mpMRI showed a sensitivity of 84.6%, a specificity of 33.3%, a PPV of 73.0%, an NPV of 50.0% and an accuracy of 68.4%. The reporting score agreement was excellent (K = 0.884, *p* < 0.001). A detailed comparison of the scoring results from reader 1 and reader 2 is reported in Table 3.

### 3.2. Agreement between mpMRI and PET-CT

#### 3.2.1. Comparison between mpMRI and Choline PET-CT

Of the 46 patients submitted to choline PET-CT, 32 had a positive mpMRI (69.6%), while only 9 (19.6%) had a positive PET-CT scan. Overall, a correlation between the results of choline PET-CT and mpMRI was found (*p* = 0.041): all patients with a positive choline PET-CT scan (9/9, 100.0%) were also positive at mpMRI; in contrast, there was a greater discordance for patients with a negative choline PET-CT, as 23/37 (62.2%) showed a positive or doubtful result at mpMRI (Table 4).

#### 3.2.2. Comparison between mpMRI and Ga-PSMA PET-CT

Among the 22 patients submitted to Ga-PSMA PET/CT, 13/22 (59.1%) reported a positive mpMRI, versus 5/22 (22.7%) showing positive results at Ga-PSMA PET-CT. The correlation between the results of Ga-PSMA PET/C and those of mpMRI was not significant (*p* = 0.360): all patients positive at Ga-PSMA PET-CT were also positive at mpMRI, except for one. A greater discordance was found for patients with negative Ga-PSMA PET-CT results, as 9/22 (52.9%) appeared positive at mpMRI (Table 5).

### 3.3. Correlation between PI-RR and PSA Level

**Figure 2:**
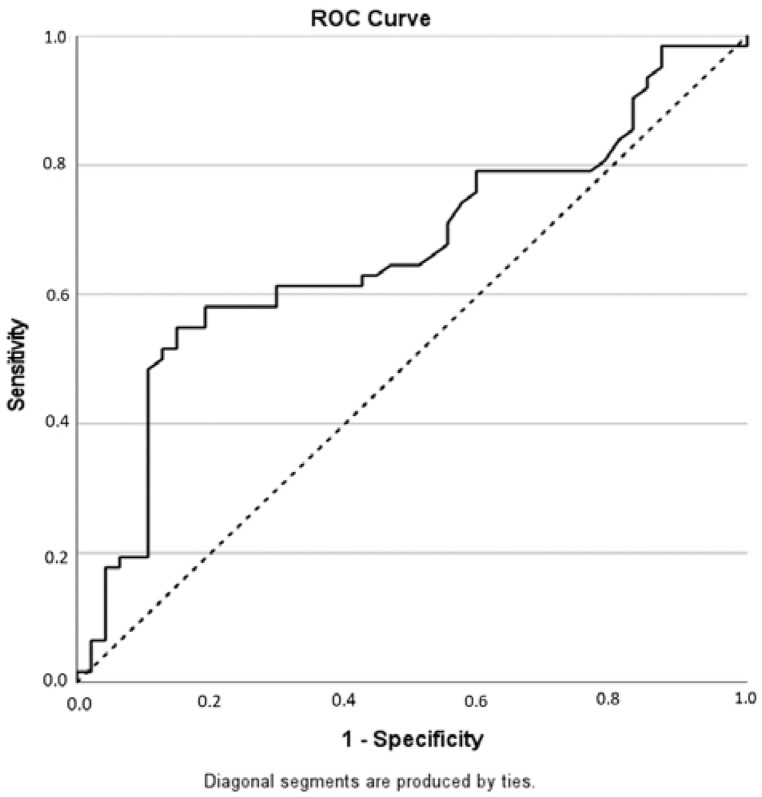
Receiver Operating Characteristic (ROC) curve for PSA predicting a PI-RR Score ≥ 3.

In 109 patients, the level of PSA was available: the mean PSA level was of 0.79 ng/mL, ranging from 0 to 5.85 ng/mL. The analysis of the PSA distribution documented an increase in the PI-RR score with an increasing PSA (ρ = 0.212, *p* = 0.027). Patients with a negative mpMRI had a mean PSA concentration of 0.53, while patients with a positive mpMRI showed a mean PSA concentration of 0.98, with a difference that was statistically significant (*p* = 0.003). the PSA level was used to predict the mpMRI result (PI-RR score ≥ 3, Figure 2).

The best cutoff to predict mpMRI positive results was 0.60 ng/mL (AUC = 0.665, 95%CI = 0.561–0.768, *p* = 0.003, sensitivity = 54.8%, specificity = 85.1%, PPV = 82.9%, NPV = 58.8%, accuracy = 67.9%) (The PSA level was <0.5 ng/mL in 57 patients; among them, 24 (42.1%) had a PI-RR score ≥ 3 at mpMRI.

## 4. Discussion

The main objective of the PI-RR is to provide guidelines to standardize image acquisition and reporting in the case of suspected local recurrence, as the PI-RADS does for the diagnosis of prostate cancer. From a technical point of view, the PI-RR proposes the same patient preparation and protocols as those described in PI-RADS version 2.1, except for recommending a T2-weighted acquisition at three orthogonal levels (axial, sagittal and coronal), including vesico-urethral anastomosis, the prostatic bed, bladder base, the periurethral tissue, the levator ani, the rectum and the residual seminal vesicles (if present) [8].

Image interpretation is primarily based on the knowledge of the post-surgical normal anatomy: MR imaging findings could include post-operative collections, retained seminal vesicles, benign prostatic remnant and post-operative fibrosis [11,12,13]. The most common sites of local recurrence are the perianastomotic site, the retrovesical region, the retained seminal vesicles [14].

**Figure 3:**
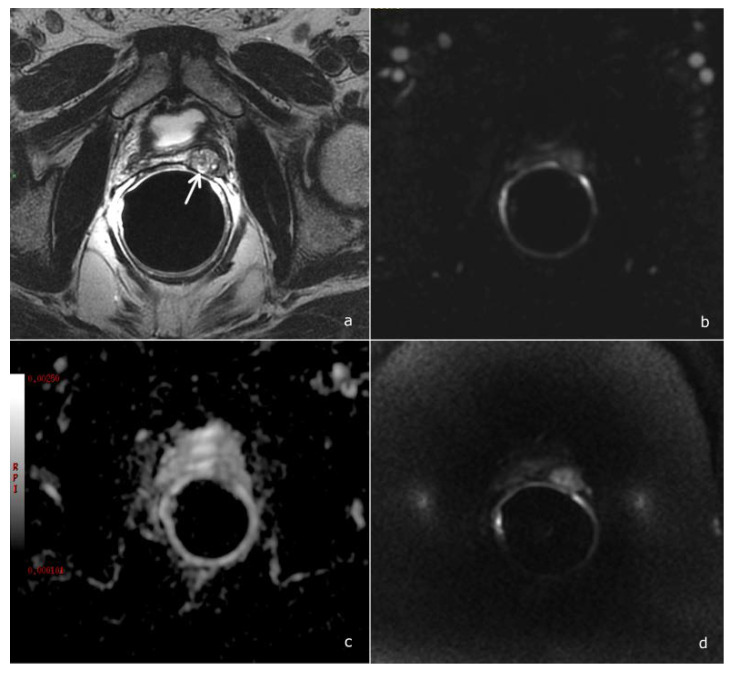
A PI-RR-score-2 patient, 68 years old, with a PSA value of 0.45 ng/mL. (**a**) T2-weighted image shows a hyperintense tissue in the rectovesical region (arrow), characterized by a homogeneous and diffuse contrast enhancement, (**b**) non-pathological signal abnormality on the ADC map, (**c**) slightly hyperintensity on DWI, (**d**) a TRUS-guided biopsy was performed, documenting benign prostatic tissue remnants.

Several studies addressed the value of mpMRI in the detection of local recurrence, pointing out the key role of perfusion-weighted imaging [15,16,17,18]. Taking into account these results, the PI-RR score was assessed considering DCE as the dominant sequence guiding the overall assessment score: at DCE imaging, local recurrence shows a rapid and early enhancement, with a kinetic different from that of post-operative fibrotic changes (which either do not enhance or enhance slowly and homogenously) [8], Figure 3.

**Figure 4:**
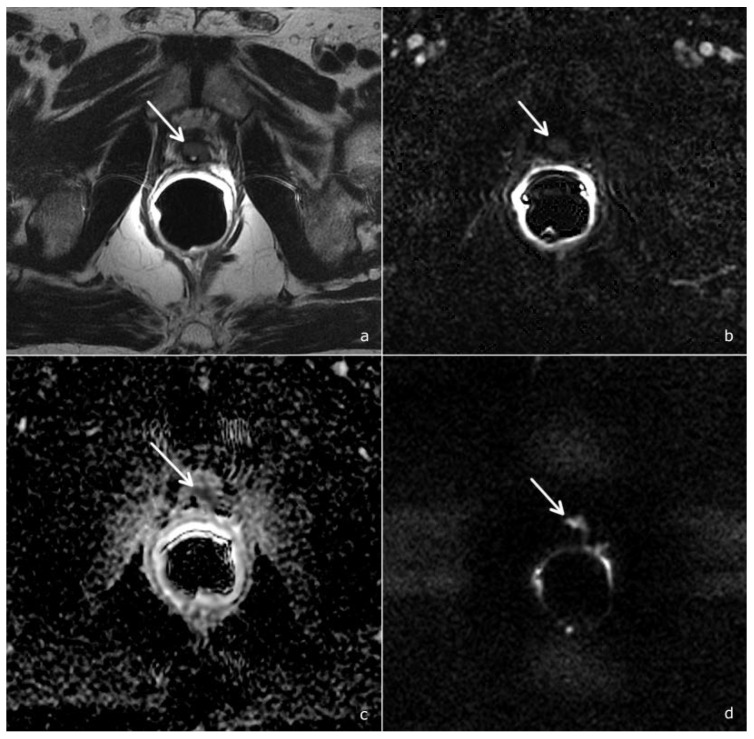
A PI-RR-score-4 (arrows), in a patient 72 years old, with a PSA value of 0.71 ng/mL. (**a**) T2-weighted image documents hypointense tissue localized anteriorly to the vesico-urethral anastomosis. (**b**) DCE depicts a focal mild and late enhancement. However, as the ADC map (**c**) and DWI (**d**) show a significant diffusion restriction (DWI score = 4), the overall PI-RR score was 4.

Also a recent study by Gaudiano et al. demonstrated that the specificity of mpMRI could be improved through the analysis of signal intensity/time curves: indeed, while benign tissue is most frequently associated with a type 1 curve, recurrences generally show type 2 and 3 curves [19]. Combining DWI to DCE imaging further increases the diagnostic accuracy of mpMRI and allows the upgrading from PI-RR 2 to PI-RR 3 and from PI-RR 3 to PI-RR 4 if the DWI score is superior or equal to 4 [8], as shown in Figure 4.

**Figure 5:**
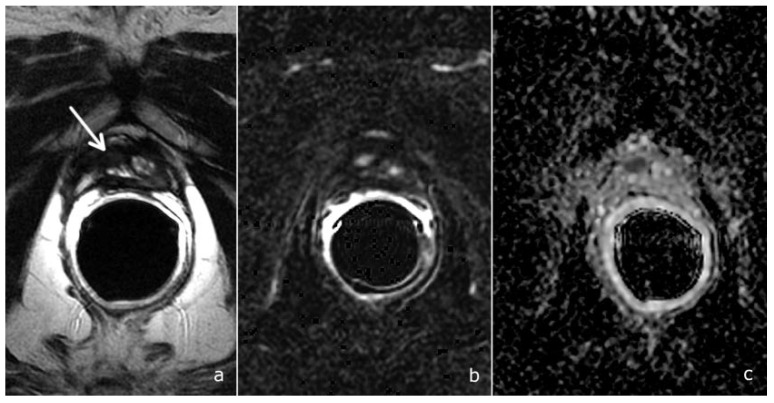
A PI-RR-score-5 patient, 70 years old, with a PSA value of 0.31 ng/mL. The patient underwent radical prostatectomy for bilateral prostate cancer, Gleason score 4 + 3, with bilateral surgical positive margins at the prostate apex. mpMRI documented a small hypointense tissue in the T2-weighted image, right-sided to the vesico-urethral anastomosis (**a**-arrow), characterized by intense and early contrast enhancement (**b**) and restricted diffusion at the ADC map, (**c**) PI-RR score = 5.

In the setting of suspected local recurrence, the main criticisms of the majority of studies are retrospective analyses, a great variability of the PSA values, a lack of a standard of reference. In most studies, the imaging results were validated using a composite reference standard consisting of heterogeneous parameters (durable PSA control after radiotherapy; concordant findings with other imaging studies; long-term follow-up; consensus achieved by a multidisciplinary board); in others, there was no reference standard, with only the “detection rate” of the imaging test reported [20]. Indeed, histological evidence of suspected local recurrence is not usually required to submit patients to salvage radiotherapy. Also in our study, histological samples were available only for a minority of patients. Nevertheless, we could evaluate the diagnostic accuracy of the PI-RR score: sensitivity and positive predictive value were high (84.6% and 73.0% respectively), while specificity and negative predictive value were low (33.3% and 50%). Overall, the accuracy was 68.4%, slightly inferior to that previously reported [9]. However, the excellent agreement among readers with different levels of experience in mpMRI confirmed the reproducibility of the PI-RR score (Figure 5).

The assessment of the PI-RR score could help in the decision-making work-up for patients with BCR. Salvage Radiotherapy has been shown to be most effective when the PSA level is <0.5 ng/mL [21]; at these low PSA values, recurrence is expected to produce a tumor with very low volume, which is extremely difficult to detect by imaging modalities; for this reason, the current practice is that patients undergo local or systemic treatment based on a clinical probability evaluation [20]. In this setting, the current European guidelines recommend a PSMA PET/CT scan in case of PSA > 0.2 ng/mL to rule out metastatic disease [1]; the PI-RR could be used as an adjunct to PSMA PET/CT for patients with high clinical suspicion of local recurrence. From the results of our study, mpMRI has a higher detection rate not only with respect to choline-PET/CT (69.6% versus 19.6%), but also with respect to PSMA PET-CT (59.1% versus 22.7%). Our results are in line with a recent study by Radzina M et al., which reported a better diagnostic accuracy of mpMRI for the detection of local recurrence, while PSMA PET/CT was superior in the detection of distant and lymph node metastases [22]. Thus, a combined use of mpMRI and PET-CT could be proposed: a PI-RR score ≥ 3 properly detects local recurrence with a high degree of certainty; in contrast, with a PI-RR score 1 or 2, PSMA PET-CT should be performed to identify distant disease and confirm the negative local findings [9].

However, the PI-RR score could have limits in the presence of low PSA values. Indeed, we observed an increase in the detection rate with increasing PSA; when PSA < 0.5, mpMRI was ≥3 for 42.1% of the patients, increasing to 54.8% when PSA ≥ 0.6. Only few studies reported a positivity rate of mpMRI at low PSA values: Counago et al. reported a positivity rate of 12.9% [23], similar to that of Liauw et al. (17.0%) [24], while Linder reported a positive rate of 94.0% for patients with PSA < 0.4 ng/mL [25].

Thus PI-RR scores of 1 and 2 do not necessary suggest a very low or low likelihood of recurrence, but may rather indicate a lower relapse volume, not detectable at mpMRI. An optimal PSA threshold justifying imaging would be interesting, as the current European Association of Urology guidelines recommend performing imaging only if the outcome influences treatment decisions and management [1]. A systematic review of imaging techniques for patients with early recurrent prostate cancer discussed the influence of the level of PSA in the performance of the imaging tests, concluding that the lower the PSA values, the less likely a scan result will be positive. At PSA < 0.5 ng/mL, the detection rate of choline PET-CT ranges from 0 to 31.3%, while the detection rate of PSMA PET/CT ranges from 11.0 to 65.0% [20]. In the attempt to optimize diagnostic appropriateness criteria, a clinical normogram to predict test positivity could be applied, as proposed by Ceci et al. [26,27]. From our results, the PI-RR score increased with the increase in the PSA level; a value of PSA ≥ 0.6 was the best cutoff to predict mpMRI positive results; however, further studies are needed to better define which kind of patients would really benefit from mpMRI. As the PI-RR score could have limits in case of low PSA values, only patients with a high probability to have a positive mpMRI should be selected, to avoid unnecessary examinations that would not impact on patients’ management.

The main limits of our study are the single-center design and the retrospective analysis, which limited the availability of a reference standard (lack of follow-up and biopsy available for 19 patients).

However, our results are consistent with those of Pecoraro et al., thus validating the diagnostic accuracy of the PI-RR score and its reproducibility through an inter-observer agreement analysis. Moreover, the detection rate of mpMRI was compared to those of choline and PSMA PET/CT.

## 5. Conclusions

The PI-RR score demonstrated an excellent inter-observer agreement supporting its wide application in the clinical practice to standardize recurrence reporting. The diagnostic accuracy was 68.4%, with the detection rate influenced by the PSA values (best cutoff to predict positive results of 0.6 ng/mL). mpMRI globally showed a higher detection rate than PET/CT scans for local recurrence.

## Figures and Tables

**Table 1:** Diffusion-weighted imaging (DWI) and dynamic-contrast enhancement (DCE) score assessment.

Sequence	Score	Pattern
**DWI**	1	No signal abnormality
	2	Diffuse moderate hyperintensity on high b-value and diffuse moderate hypointensity on the ADC map
	3	Focal marked hyperintensity on high b-value or focal marked hypointensity on the ADC map
	4	Focal marked hyperintensity on high b-value and focal marked hypointensity on the ADC map, not on the same site as that of the primary tumour, or tumour site not known
	5	Focal marked hyperintensity on high b-value and focal marked hypointensity on the ADC map, on the same site as that of the primary tumour
**DCE**	1	No enhancement
	2	Diffuse enhancement
	3	Focal late enhancement
	4	Focal early enhancement, not on the same site as that of the primary tumour, or tumour site not known
	5	Focal early enhancement, on the same site as that of the primary tumour

**Table 2:** PI-RR score distribution in relation to the biopsy results.

PI-RR	Biopsy Result		Total
			(N = 19)
	Negative	Positive	
**1**	0	1	1
**2**	2	1	3
**3**	1	3	4
**4**	2	7	9
**5**	1	1	2

**Table 3:** Scoring agreement between reader 1 and reader 2.

PI-RR	Reader 2					Total
						(N = 134)
Reader 1	1	2	3	4	5	
**1**	42	0	0	0	0	42
	−100.00%	0.00%	0.00%	0.00%	0.00%	
**2**	0	17	3	0	0	20
	0.00%	−85.00%	−15.00%	0.00%	0.00%	
**3**	0	0	15	2	0	17
	0.00%	0.00%	−88.20%	−11.80%	0.00%	
**4**	0	0	6	31	0	37
	0.00%	0.00%	−16.20%	83.8%	0.00%	
**5**	0	0	1	0	17	18
	0.00%	0.00%	−5.60%	0.00%	−94.40%	
**Total**	42	17	25	33	17	134

**Table 4:** Comparison between mpMRI and Choline PET-CT.

CHOLINE PET-CT	mpMRI		Total
			(N = 46)
	Negative	Positive	
	N (%)	N (%)	
**Negative**	14	23	37
	−37.80%	−62.20%	
**Positive**	0	9	9
	0.00%	−100%	

**Table 5:** Comparison between mpMRI and Ga-PSMA PET-CT.

Ga-PSMA PET-CT	mpMRI		Total
			(N = 22)
	Negative	Positive	
	N (%)	N (%)	
**Negative**	8	9	17
	−47.10%	−52.90%	
**Positive**	1	4	5
	−20.00%	−80.00%	

## Data Availability

The data were collected on an anonymous database. The datasets analyzed during this study are available from the corresponding author upon reasonable request.
